# Signatures of selection in five Italian cattle breeds detected by a 54K SNP panel

**DOI:** 10.1007/s11033-013-2940-5

**Published:** 2014-01-19

**Authors:** Giordano Mancini, Maria Gargani, Giovanni Chillemi, Ezequiel Luis Nicolazzi, Paolo Ajmone Marsan, Alessio Valentini, Lorraine Pariset

**Affiliations:** 1 Department for Innovation in Biological, Agro-food and Forest Systems, University of Tuscia, 01100 Viterbo, Italy; 2 CASPUR Inter-University Consortium for the Application of Super-Computing for Universities and Research, Rome, Italy; 3 Institute of Zootechnics, Sacro Cuore Catholic University, Piacenza, Italy

**Keywords:** Selection signatures, SNP, Cattle breeds, MDS, Bayesian assignment

## Abstract

In this study we used a medium density panel of SNP markers to perform population genetic analysis in five Italian cattle breeds. The BovineSNP50 BeadChip was used to genotype a total of 2,935 bulls of Piedmontese, Marchigiana, Italian Holstein, Italian Brown and Italian Pezzata Rossa breeds. To determine a genome-wide pattern of positive selection we mapped the *F*_*st*_ values against genome location. The highest *F*_*st*_ peaks were obtained on BTA6 and BTA13 where some candidate genes are located. We identified selection signatures peculiar of each breed which suggest selection for genes involved in milk or meat traits. The genetic structure was investigated by using a multidimensional scaling of the genetic distance matrix and a Bayesian approach implemented in the STRUCTURE software. The genotyping data showed a clear partitioning of the cattle genetic diversity into distinct breeds if a number of clusters equal to the number of populations were given. Assuming a lower number of clusters beef breeds group together. Both methods showed all five breeds separated in well defined clusters and the Bayesian approach assigned individuals to the breed of origin. The work is of interest not only because it enriches the knowledge on the process of evolution but also because the results generated could have implications for selective breeding programs.

**Electronic supplementary material:**

The online version of this article (doi:10.1007/s11033-013-2940-5) contains supplementary material, which is available to authorized users.

## Introduction

Present day cattle breeds are the result of years of human selection, adaptation to different environments and demographic effects as domestication, migration and selection, all contributing to the actual patterns of genetic diversity [1, 2]. During the domestication process, breeds were selected for productivity traits as, for example, milk yield [3]. Moreover, animal and semen exchange, carried out to improve production characteristics, have affected the genetic features of the breeds. This anthropic selection has influenced the genetic structure of cattle breeds, therefore a high percentage of loci purposely chosen for influencing potentially selected traits could result under selection [4, 5].

Recently, the availability of high density SNP panels has given the possibility of performing population genetic studies in cattle populations using thousands of markers distributed across the entire genome. Medium density SNP panels have been used for example to analyze the genetic structure of cattle populations [6–8] to study past effective population size [9], to detect selection signatures [10], and to discover copy number variation (CNV) suitable for understanding genetic features and accelerating genetic improvement for complex traits [11].

We considered a total of 2,935 bulls belonging to two dairy (Italian Brown and Italian Holstein), two beef (Piedmontese and Marchigiana) and one double purpose (Italian Pezzata Rossa) breeds. The Italian Holstein derives from Dutch and North America Holstein breeds imported in Italy in the late XX century and it is currently the most common dairy breed. Piedmontese is mainly located in Northern Italy and it was in the past a dual purpose breed, while today it is selected for beef traits mainly exploiting a private myostatin mutation [12]. Marchigiana is a beef breed from central Italy derived from very ancient breeds like Chianina and Romagnola breeds. The Italian Brown was originally a multi purpose breed reared in the Alps; it was selected from 1950 as a dairy breed by importing Swiss Brown bulls from the U.S. Pezzata Rossa, Simmental, is a beef/dairy breed imported from Swiss/Austria and herded mostly in North East Italy.

The aim of this study was to identify genomic regions potentially under selection in the above five Italian cattle breeds using a 54K medium-density SNP panel. Our results could have implications for selective breeding programs by identifying signatures of artificial selection in gene involved in milk, meat or functional traits. We analyzed also the genetic structure of the breeds by classical multidimensional scaling and by Bayesian inference methods.

## Materials and methods

### Samples and high throughput genotyping

The initial sample was formed by 2,935 bulls: 761 Italian Brown, 899 Italian Holstein, 323 Piedmontese, 464 Marchigiana and 488 Italian Pezzata Rossa bulls, respectively. The sample represents almost all the bulls available in Italy for all breeds but Holstein, where the bulls analyzed correspond to slightly less than a half of the available ones. Genomic DNA was extracted from semen using the NucleoSpin Tissue kit (Macherey-Nagel) according to manufacturer’s instruction. DNA was checked for quality on agarose gel and quantified using a DTX microplate reader (Beckman Coulter) after staining with Picogreen (Invitrogen). Samples were genotyped using BovineSNP50 Genotyping BeadChips (Illumina, San Diego, CA, USA). Genotyping was outsourced to Geneseek (www.geneseek.com). The 50K SNP array contains 54,001 SNPs distributed across the entire genome, with an average SNP spacing of 51 Kb and a proportion of known chromosome positions of about 97 %; SNP positions within each chromosome were based on the *Bos taurus* genome assembly Btau_4.0 [13].

### Data editing and genome-wide analysis

Data were initially filtered using the GenABEL R package (http://www.r-project.org, http://mga.bionet.nsc.ru/~yurii/ABEL/GenABEL/). Only autosomal markers were used and SNP with complete map information were used. Sires and markers with a call rate under 99 % were discarded as well as SNPs having a minor allele frequency (MAF) <5 % according to the currently employed thresholds [14]. Sires were checked for abnormally high autosomal heterozygosity and discarded when showing a false discovery rate (FDR) <1 % [15]. Then, sires of each breed were separated and Hardy–Weinberg equilibrium (HWE) was checked within each breed setting a threshold of *P* < 0.01 in the filtered data set [16]. Finally, data were pooled again and filtering criteria explained above were applied once more. Kinship among sires was estimated directly from genomic data as proposed by Astle and Balding [17].

To determine a genome-wide pattern of positive selection, the *F*_*st*_ at each locus was calculated [18]. The loci under selection are expected to show an allele frequency that deviate from that of neutral loci, leading to an increased level of genetic differentiation. *F*_*st*_ values were then plotted against genome location. Signatures of selection can be recognized when adjacent SNPs in a region show high *F*_*st*_ [19] thus we used a sliding window approach, with a window of eight SNPs. A region with high *F*_*st*_ implies divergent selection between breeds, whereas low *F*_*st*_ imply balancing selection between breeds. Fixation index was calculated using the method proposed by Nei and Chesser [20] using in-house written R codes; this method was chosen because the sample includes (almost) all sires in the national herdbooks for three breeds (Marchigiana, Piedmontese and Pezzata Rossa) and thus fixed effects errors of sampling, i.e. effects unbounded by a prior distribution, seemed more important. Graphs were obtained using matplotlib (http://matplotlib.sourceforge.net/).

Genetic distance between breeds and sires was estimated calculating the matrix *d*_*ij*_ = (0.5 − *k*_*ij*_) where *d* is the distance and k is genetic kinship coefficient for sires *i* and *j* and then applying classical multidimensional scaling to the complete matrix. STRUCTURE software v. 2.3.2.1 [21] was used to analyse population structure. A total of 15,000 Markov chain Monte Carlo (MCMC) iterations (5,000 burn-in and 10,000 sampling) were performed for each tested K using the admixture model, considering allele frequencies correlated among populations and including no informative prior about individual membership; K values from 2 to 5 were used. Five independent runs for each tested K value were performed. The number of steps was chosen following [22], although for each K a single run of 50,000 iterations to test the effects of longer runs was performed. Evanno et al. [23] reported that in most cases, the estimated ‘log probability of data’ did not provide a correct estimation of cluster number (K value), and argued that an ad hoc statistic ΔK based on the rate of change in the log probability of data between successive K values could accurately detect true K. The suggested statistics was:

$$\varDelta K = m(|L\left( {K + 1} \right) - 2L\left( K \right) + L\left( {K - 1} \right)|)/s\left[ {L\left( K \right)} \right],$$

where *L(K)* represents the *K*th *LnP(D)*, *m* is to the mean of 10 runs and *s* their standard deviation. We used the method of Evanno et al. [23] to estimate the number of populations. Graphical visualization of STRUCTURE results was performed by means of the DISTRUCT package [24].

## Results

### Data editing

Elimination of markers on the X chromosome or with incomplete map information left 51,515 SNPs out of the 54,001. A total of 138 sires and 12,388 markers showing a call rate <99 % were excluded. Additional 8,874 SNPs with a MAF <5 % were discarded. Then, a total of 1,443 markers were discarded because they were out of HWE in at least one breed. No sires or markers were excluded after the second check on call rates and allele frequencies. The final complete dataset was thus formed by 2,797 sires (755 Italian Brown, 861 Italian Holstein, 483 Italian Pezzata Rossa, 317 Piedmontese and 381 Marchigiana bulls) and 29,848 SNPs.

### Selection signatures

**Fig. 1:**
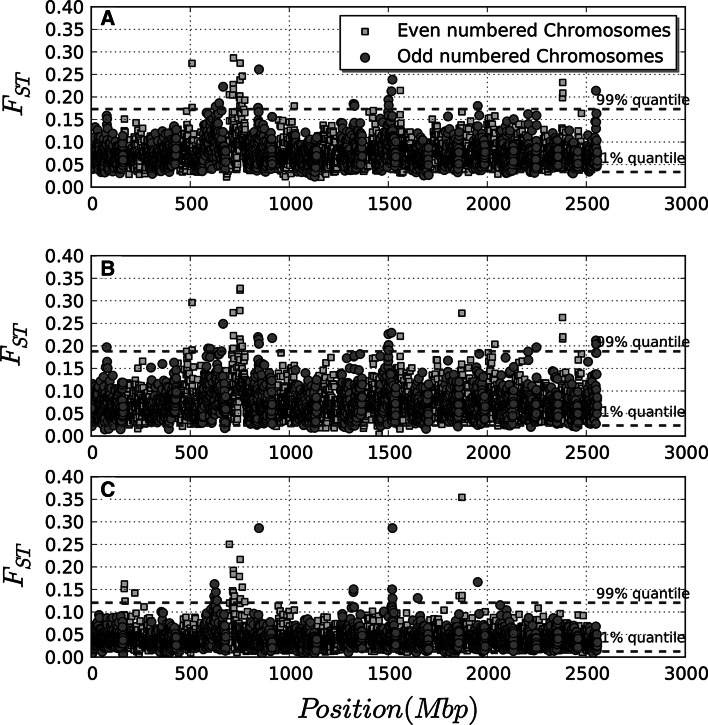
Genome wide Manhattan plots of fixation index (*F*_*st*_) calculated for all polymorphisms averaged over a window of 8 SNPs within each chromosome and calculated for all five breeds (**a**), dairy breeds (**b**) and beef breeds (**c**). *F*_*st*_ for odd and even chromosomes are represented with blue circles and red squares, respectively. The values corresponding to the lowest 1 and 99 % quantiles are represented with *green dashed lines*. (Color figure online)

**Fig. 2:**
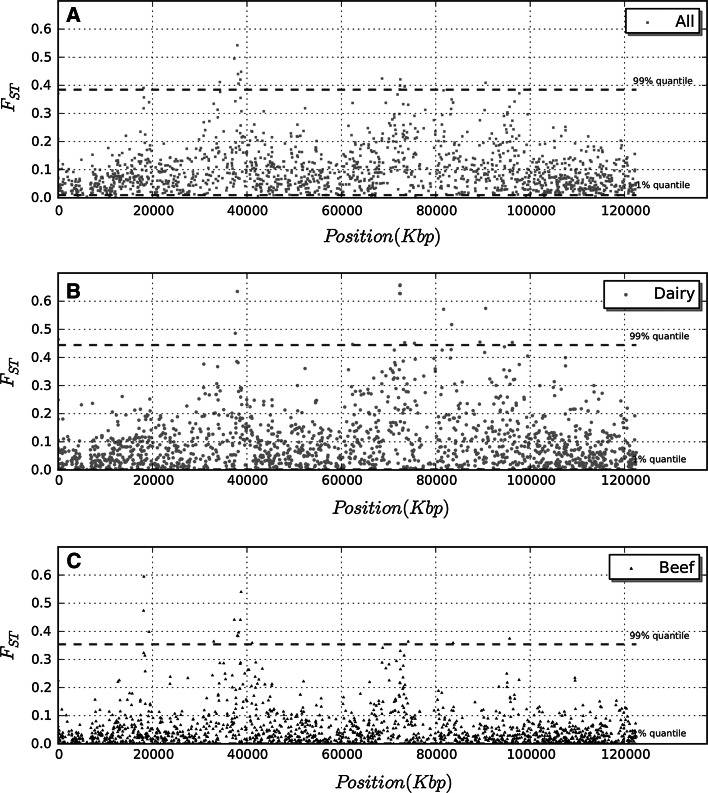
Plot of fixation index (*F*_*st*_ *)* calculated for all markers on BTA6 averaged over a 3 SNP window within all breeds (**a**), dairy breeds (**b**) and beef breeds (**c**). The values corresponding to the lowest 1 and 99 % quantiles are represented with *green dashed lines*. (Color figure online)

Figure 1 shows *F*_*st*_ values calculated for all autosomal SNPs and averaged over a 8-wide SNP window within each chromosome. Results including all five populations, only dairy breeds and only beef breeds are displayed in panels A, B and C, respectively. Fixation index values containing the 98 % of point are also indicated with green lines. In panel A, *F*_*st*_ ranges from 0.04 to 0.30, with an average value of 0.077 ± 0.058, with the exception of a single *F*_*st*_= 0 signal on BTA 19. SNP clusters with *F*_*st*_ values above 0.0175 (the 99 % threshold) were located on chromosomes 3–6, 13, 14, 16, 18, 26 and 29. In most cases *F*_*st*_ peaks are formed by only one or two groups of 8 markers, therefore spanning about 4 Mb. More consistent signals are present on BTA13 and on BTA6, where peaks are formed by 11 and 4 groups of markers above the 99 % quantile value. A similar pattern is shown in Panels B and C, where *F*_*st*_ values are calculated for dairy and beef breeds separately; the strongest signals are still observed on BTA6. *F*_*st*_ obtained in dairy breeds are higher than those observed for beef breeds. The peak identified in dairy breeds spans from 72.35 to 72.47 Mb on BTA6 (Figs. 1b, 2b). Three of the four SNPs identified in this region are located in the platelet-derived growth factor receptor, alpha polypeptide (PDGFRA) gene involved in the reproduction process and in the regulation of calcium level and near the KIT (gene v-kit Hardy–Zuckerman 4 feline sarcoma viral oncogene homolog) gene expressed in the lactating bovine mammary gland and implicated in determining coat colour [5]. In dairy breeds a weaker peak, formed by four markers, is also observable around 38 Mb on BTA6 where ATP-binding cassette, sub-family G (ABCG2) and polycystic kidney disease 2 (PKD2) genes are located. The two genes play a role in the regulation of bovine lactation [25] and in calcium homeostasis, respectively [26].

In beef breeds the peak on BTA6 consists of six SNPs spanning from 37.32 to 38.76 Mb (Figs. 1c, 2c). This interval contains 15 genes including LAP3 (leucine aminopeptidase 3) non-SMC condensin I complex subunit G (NCAPG) and ligand-dependent nuclear receptor corepressor-like protein (LCORL), genes involved in calving ease [27].

Figure 2a shows *F*_*st*_ values for markers on BTA6 only, without any averaging. Several markers that have *F*_*st*_ above the 99 % quantile can be observed at 18 Mb, between 36 and 39 Mb and at 95 Mb, but the strongest signal (considering either the number of SNPs with high *F*_*st*_ or the maximum value) can be observed at ~38 Mb.

**Fig. 3:**
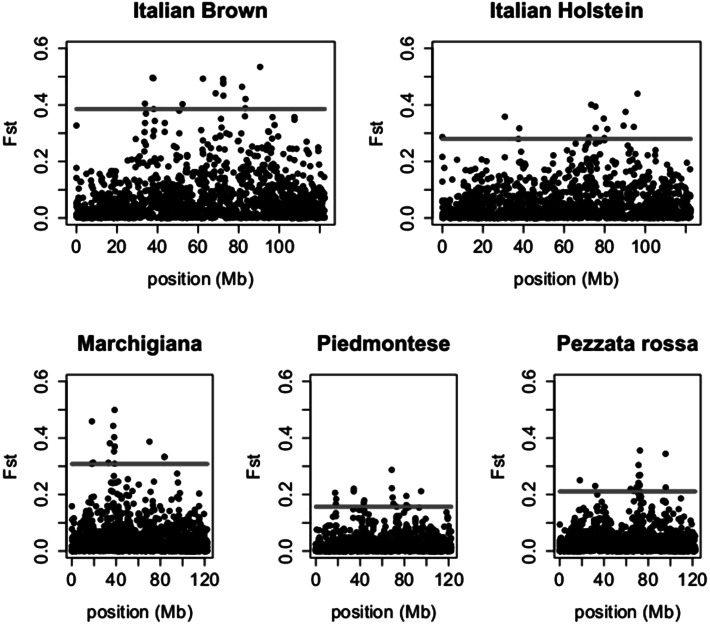
Plot of *F*_*st*_ values calculated on BTA6 for each breed versus all others. The values corresponding to the 99 % quantiles are represented with *red lines*. (Color figure online)

Moreover we calculated the *F*_*st*_ values for BTA6 comparing each breed against the remaining four (Fig. 3). In Italian Brown breed we can observe a signals at 72 and 37 Mb, in Italian Holstein and Pezzata Rossa breeds the strongest signal can be observed at 72 Mb, while in Marchigiana a peak is located around 37 Mb. The Piedmontese breed showed two signals one around 18 Mb and the other around 68 Mb.

The *F*_*st*_ calculated only for dairy breeds (Fig. 1b) shows a peak formed by four SNPs spanning from 47.23 to 48.30 Mb on BTA13. In this region we identified 14 genes among which CDS2 (CDP–diacylglycerol synthase) could affect milk fat composition. Four significant SNPs on chromosome 13 at positions 67197635, 67464116, 67490718, 67766784 were identified in beef breeds (Fig. 1c). Of the 8 genes in this region, SRC (v-src sarcoma viral oncogene homolog avian**)** and CTNNBL1 (catenin, beta like 1) may be related to muscle formation and to body weight, respectively.

### Multidimensional scaling

**Fig. 4:**
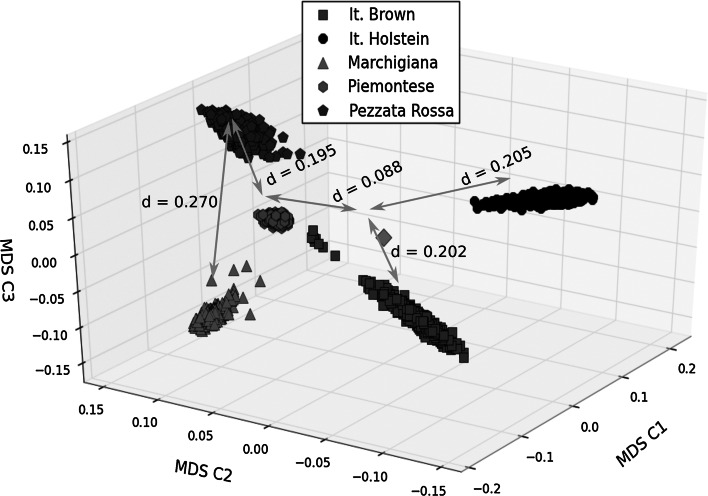
Classical multidimensional scaling plot of genomic distance calculated as 0.5—genomic kinship for all five breeds. The first three components are shown as C1, C2 and C3, respectively. Subjects are depicted as *grey squares* (Italian Brown), *black circles* (Italian Holstein), *red hexagons* (Italian Pezzata Rossa), *blue triangles* (Marchigiana), *green pentagons* (Piedmontese), respectively. The centre of mass of the complete distribution is represented as a *magenta diamond* and relative distances between each cluster centre or between any cluster and the general centre is indicated by *cyan arrows* and annotated in the figure. (Color figure online)

Figure 4 shows the first three components of the multidimensional scaling decomposition of the genetic distance matrix (calculated as 0.5-kinship). Relative distances between selected clusters are shown in the graph and range from 0.195 (Piedmontese–Pezzata Rossa) to 0.291 (Marchigiana–Holstein). The five breeds form compact clusters separated from each other with the partial exception of a small group of outlying Brown bulls located near the “centre of mass” of clusters (represented with a diamond). The first component (C1 hereafter) separates Brown and Holstein from Piedmontese, Marchigiana; Pezzata Rossa is located between these two breeds; Brown and Holstein are both located at comparable distance from the centre on the negative half of component two (C2 hereafter) and separated from the other breeds; both clusters form approximate ellipsoids with the major axis along different diagonals in the plane of the first two components while component three (C3 hereafter) contribution is very small. The opposite is true for the relative position of beef breeds and Pezzata Rossa for which the greatest differentiation is due to C3. Piedmontese forms the most compact cluster and is closer to the centre. Also, it must be observed that, although Pezzata Rossa is a double purpose breed, its cluster is actually more distant from dairy breeds as compared to Marchigiana and Piedmontese and that Piedmontese is more distant from Marchigiana than from Pezzata Rossa. Figures S1 and S2 in the Supplementary materials show analogous calculations restricted to either dairy or beef breeds. In both cases two breeds (Brown and Pezzata Rossa in Fig. S1, Marchigiana and Piedmontese in Fig. S2) are located in the C1–C2 plane while the remaining population (Holstein in Fig. S1 and Pezzata Rossa in Fig. S2) is located in a different quadrant in the C1–C2 plane and is highly dispersed along C3.

### Bayesian inference

**Fig. 5:**
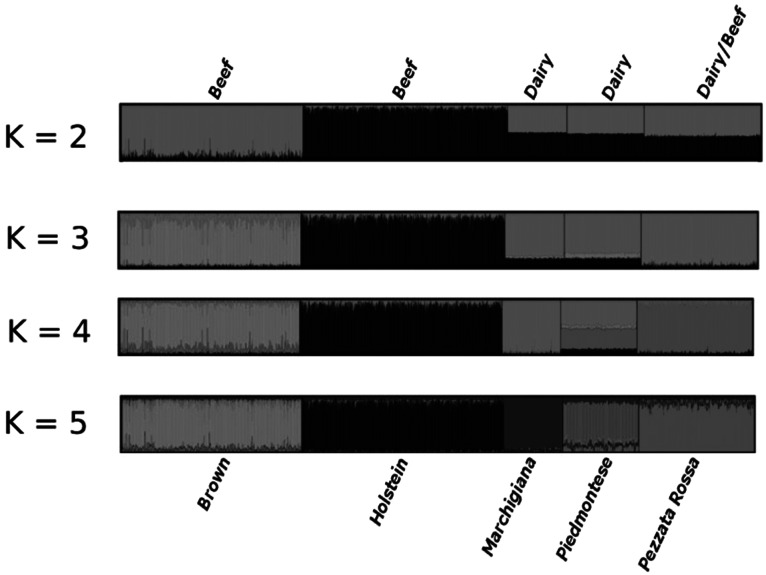
Summary plot of Q estimates (estimated membership coefficients for each individual, in each cluster) for K = 2, 3, 4, 5 obtained with a 5,000 burn-in, and 35,000 MCMC, under the admixture model, for the breeds analyzed. Individuals are represented in breed groups separated by *vertical lines*. Breed is indicated above while the breeds’ attitude is indicated under each group. Each individual is represented by a *single vertical line* broken into K coloured segments, with lengths proportional to each of the K inferred clusters. *Each colour* represents the proportion of membership (M) of each individual (represented by a *vertical line*) to the K clusters. (Color figure online)

To estimate the number of genetic clusters within the 2,797 cattle samples and 29,848 SNPs, a parametric genetic mixture analysis implemented in the STRUCTURE software was performed. Between 2 and 5 clusters (K values) were tested using the admixture model, considering allele frequencies correlated. Consistent results across runs were obtained and a clear clustering of breeds was observed for any K tested (Fig. 5). With K = 2 Brown and Holstein individuals are assigned to different clusters while sires of the other three breeds belong to each cluster with probabilities near 50 %. The separation becomes sharper when three clusters are hypothesized: in this case Marchigiana, Piedmontese and Pezzata Rossa sires are assigned to a third cluster different from Brown and Holstein (although some noise is present for Piedmontese. With K = 4 Marchigiana also forms a distinct cluster while Piedmontese is assigned with comparable probability to either Marchigiana or Pezzata Rossa and more unlikely to Brown or Holstein (the noise present for K = 3 is basically conserved). Finally, with K = 5 Piedmontese is assigned to a distinct cluster. Under the ad hoc criterion of Evanno et al. [23] K = 5 was the most likely number of genetic groups.

## Discussion

Recently many innovative tools, such as medium or high density SNP chips, have been developed for various domesticated species. In this study we presented two applications of population genetic analysis in five Italian cattle breeds using 50K bovine SNP chips. We first investigated the potential of SNP markers to identify selection signatures peculiar of each breed and then we analyzed the genetic structure in the same samples.

By mapping the *F*_*st*_ values against genome location we identified genes showing signatures of positive selection involved in biological processes such as reproduction, metabolism of lipids, calving ease. A strong selection signal was observed on BTA6 when considering *F*_*st*_ across all cattle breeds. Interestingly, the *F*_*st*_ calculated only in dairy breeds revealed the evidence for selection in the region located at 72.45 Mb on chromosome 6, far from the caseins cluster, for which selection is carried out in some breeds. The peak is located near the PDGFRA gene which is associated with β-estradiol and implicated in the reproduction process and in the regulation of calcium level and near the KIT gene expressed in the lactating bovine mammary gland and implicated also in determining coat colour. These results are consistent with those obtained by Flori et al. [28] and Stella et al. [5] who found a positive selection signature in the same region in dairy cattle breeds. Flori et al. [28] used the *F*_*st*_ approach to detect the selection signatures in three French dairy cattle breeds and highlighted 13 significant signatures including the PDGFRA gene which is proposed as candidate gene. Stella et al. [5] reported the largest composite log likelihood (CLL) in the same location on BTA6 within the KIT gene which is responsible for the piebald phenotype in four of the five dairy breeds analyzed. The peak around 38 Mb falls near the ABCG2 and PKD2 genes. Several studies identified a QTL affecting milk yield and milk composition on chromosome 6 in a region around 38 Mb containing ABCG2 gene [25, 29, 30]

Recently the results obtained by Wei et al. [25] suggested that ABCG2 plays a role in mammary epithelial cell proliferation and that the polymorphisms in this gene may influence milk production.

The other interesting gene in the region is *PKD2* gene that could be related with the content of water in the milk since it is involved in calcium homeostasis [26].

The peak located at ~38 Mb on BTA6 in beef breeds is near LAP3, NCAPG and LCORL. LAP3 encodes for a leucine aminopeptidase, which is responsible of the oxytocin hydrolysis [31]. Recent studies demonstrated the role of LAP3 in calving ease in Norwegian Red cattle [32]. Moreover, Bongiorni et al. [27] found a strong association between LAP3, NCAPG, LCORL and calving ease trait in Piedmontese.

These results are in agreement with the *F*_*st*_ values calculated for each breed against the other in BTA6.

The signal in dairy breeds is due mainly by Bruna, Holstein and Pezzata Rossa, while the signal around 37 Mb identified in beef breeds is due mainly to Marchigiana breed (Fig. 3). It is worth to notice that the Piedmontese breed shows a peak spanning between 17 and 18 Mb on BTA6. This region contains five genes including COL25A1 collagen gene.

A cluster of signals reflecting strong evidence of selection was observed also in BTA13. When we analyzed separately the *F*_*st*_ for beef breeds (Fig. 1c) a strong peak at position 67 Mb could be observed. Two interesting genes are located in this region: SRC and CTNNBL1. SRC is involved in the regulation of actin cytoskeleton and in the focal adhesion pathway [33]. Some studies reported the role of focal adhesion pathway for muscle formation in cattle [34] and muscle strength and integrity in racing horses [35]. The other gene, CTNNBL1, is associated with body weight and height [36]. In human it has been showed to be involved in the W_nt_/beta-catenin-signaling pathway and associated with obesity [36]. In dairy breeds we observed a peak spanning 47–48 Mb on BTA13 near the CDS2 gene which could influence milk fat composition; the gene is in fact involved in the phospolipid biosynthetic process. The Table 1 shows a list of candidate genes for genomic regions presenting the most extreme peaks in dairy and beef breeds.

**Table 1:** List of candidate genes for genomic regions presenting the most extreme peaks in dairy and beef breeds

BTA	Start in Mb	End in Mb	Candidate gene	Gene name and function
Dairy				
6	37.52	38.1	ABCG2	ATP-binding cassette, sub-family G
			PKD2	Polycystic kidney disease 2
6	72.35	72.47	PDGFRA	Platelet-derived growth factor receptor, alpha polypeptide
			KIT	v-kit Hardy–Zuckerman 4 feline sarcoma viral oncogene homolog
13	47.23	48.3	CDS2	CDP–diacylglycerol synthase
Beef				
6	37.00	38.76	LAP3	Leucine aminopeptidase 3
			NCAPG	Non-SMC condensin I complex subunit G
			LCORL	Ligand-dependent nuclear receptor corepressor-like protein
13	67.19	67.76	SRC	v-src sarcoma viral oncogene homolog avian
			CTNNBL1	Catenin, beta like 1

No *F*_*st*_ peaks have been detected on or near the few genes today known to influence dairy or beef traits, like DGAT1, caseins, myostatin, leptin. This may be due to the large genetic network that influences the complex traits under selection, as well as the changes of the selection policies cross time: i.e. at least in Italy and many European countries, in the early days, the main selection objective was milk yield, afterwards it was protein and fat percentage, now sustainability traits are included in the selection index.

Regarding the assessment of the genetic structure we used Bayesian and multidimensional scaling approaches. Multidimensional scaling separated each breed in five well defined clusters. Piedmontese formed the most compact cluster indicating that the breeding policy in this breed tends towards a narrower genetic basis. It has to be noted that in the past 50 years the selection has been oriented to strongly select for the double muscling trait and culling all non carrier subjects. It is worth to observe that the double purpose Pezzata Rossa is more distant from dairy breeds than beef breeds, suggesting in this breed a different management of selection and lack of admixture with other dairy breeds. Interesting, the Italian Brown showed a small group of outlying bulls in the MDS plot suggesting a potential substructure maybe due to the double type of exploitation of this breed both in high producing farms in the valleys and in harsher conditions in the mountains.

The genetic isolation and lack of admixture among the two dairy breeds are confirmed by Bayesian analysis in which the breeds do not cluster on the basis of their purpose even for K = 2 (Fig. 5). This means that the differentiation pre-dates the selection for different purposes. Beef and dual purpose breeds tend to cluster together up to K = 3 and at K = 4 Piedmontese is assigned almost equally to the other two beef breeds. We hypothesize in this case a possible convergent artificial selection for beef breeds.

## Electronic supplementary material

Below is the link to the electronic supplementary material.

Supplementary material 1 (DOC 228 kb)
