# Supplementary material for: Signatures of selection in five Italian cattle breeds detected by a 54K SNP panel
Source: Mol Biol Rep. 2014 Jan 19;41(2):957–65. doi: 10.1007/s11033-013-2940-5 (PMC3929051; doi:10.1007/s11033-013-2940-5)

### Signature of selection in five Italian cattle breeds detected by a 54k density SNP panel

### Giordano Mancini^1,2^, Maria Gargani^1§^, Giovanni Chillemi^2^, Ezequiel Luis Nicolazzi^3^, Paolo Ajmone Marsan^3^, Alessio Valentini^1^, Lorraine Pariset^1^

**Figure S1**. Classical Multidimensional Scaling plot of genetic distance calculated as genomic distance calculated as 0.5 – genomic kinship for dairy breeds. The first three components are shown as C1, C2 and C3, respectively. Subjects are represented as grey squares (Italian Brown), black circles (Italian Holstein), red hexagons (Italian Pezzata Rossa). The center of mass of the complete distribution is represented as a magenta diamond.


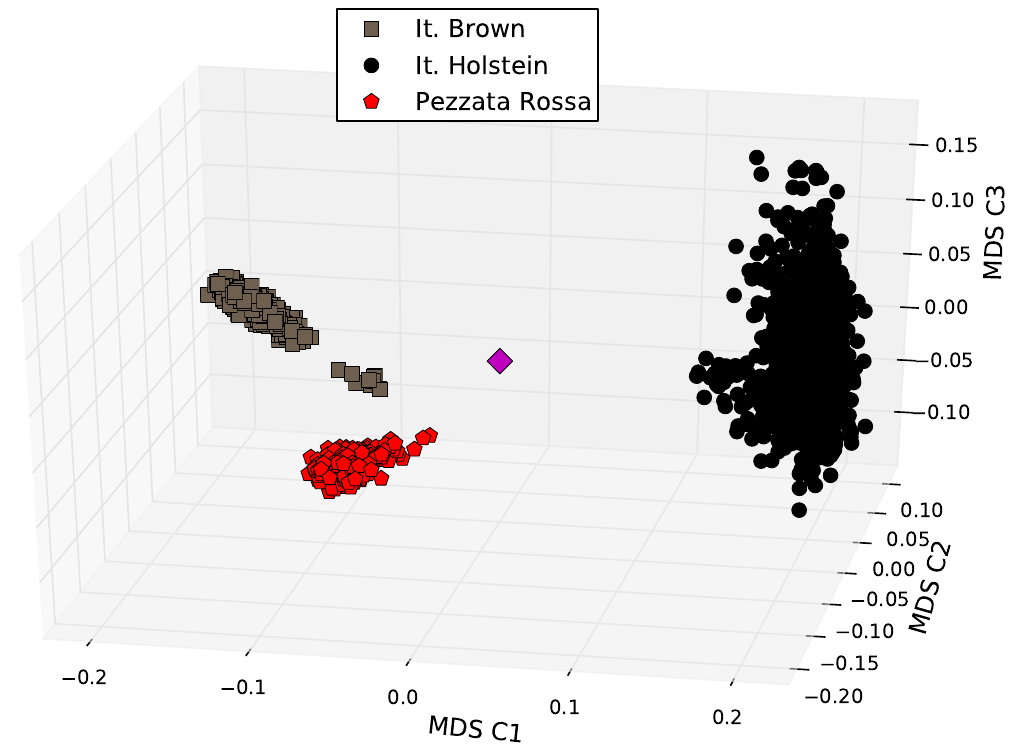


**Figure S2.** Classical Multidimensional Scaling plot of genetic distance calculated as genomic distance calculated as 0.5 – genomic kinship for beef breeds. The first three components are shown as C1, C2 and C3, respectively. Subjects are depicted as red hexagons (Italian Pezzata Rossa), blue triangles (Marchigiana), green pentagons (Piedmontese). The center of mass of the complete distribution is represented as a magenta diamond.


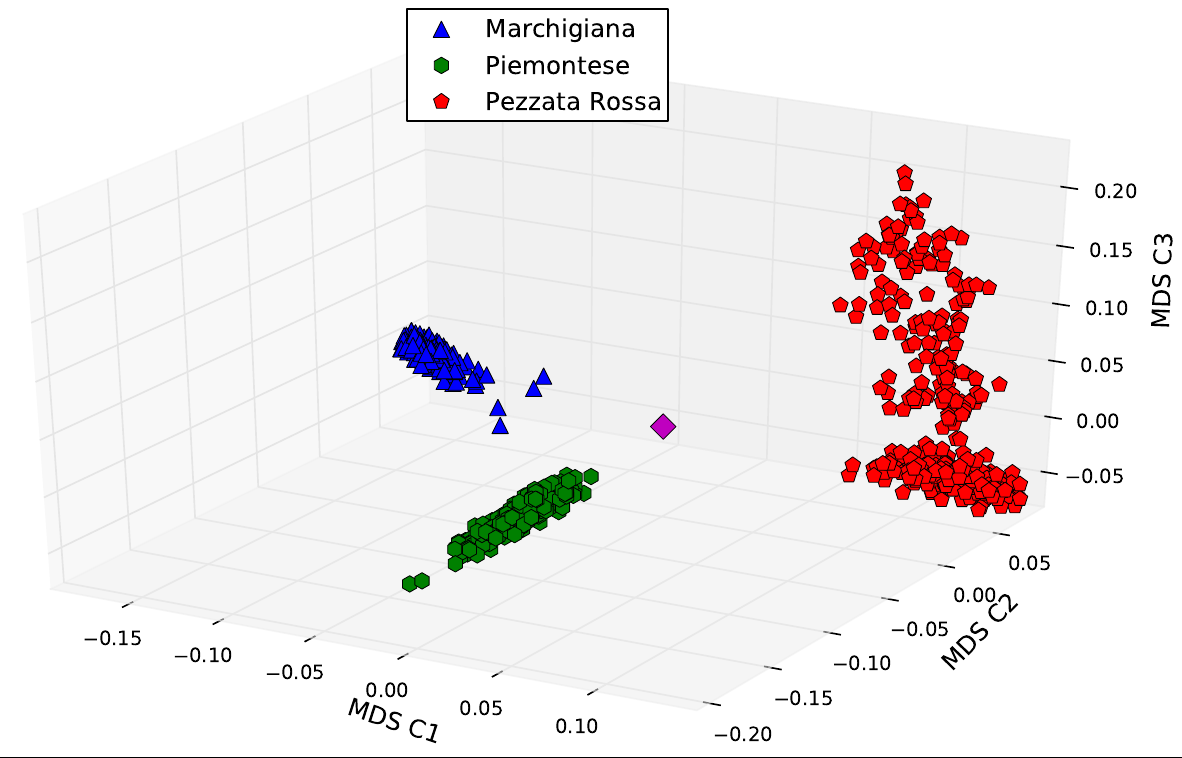

Supplement: Supplementary file 1 — Supplementary material 1 (DOC 228 kb) [file 11033_2013_2940_MOESM1_ESM.doc]
